# Modification of atrioventricular node in a special condition treating paroxysmal supraventricular tachycardia

**DOI:** 10.4103/0975-3583.74266

**Published:** 2010

**Authors:** Jun-Hua Wang, Peng Zhou, Yu-Qian Li, Jin-Jin Sun, Wei-Jie Tan, Cong-Chun Huang, Xin-Ya Yu, Chao-Zhong Liu, Hui-Lan Luo

**Affiliations:** *Department of Cardiology, Air Force General Hospital, PLA, No.30, Fucheng Road, Haidian District, Beijing, PR, China*; 1 *Section on Cardiology, Internal Medicine, Wake Forest University School of Medicine, Medical Center Boulevard, Winston-Salem, NC, USA.*

**Keywords:** Atrioventricular nodal reentrant tachycardia, paroxysmal supraventricular tachycardia, radiofrequency catheter ablation

## Abstract

Modification of atrioventricular node is a usual and necessary operation to cure atrioventricular nodal reentrant tachycardia (AVNRT). In this operation, atrioventricular block is the most severe complication and its prevention is of our great concern. This complication always occurs under some special circumstances with potential risk. So, it is very important to realize such conditions, as in this paper. A patient with paroxysmal palpitation for 10 years, aggravating to shortness of breath with chest distress for 1 year; cardiac electrophysiological examination found slow conduction in both antegrade and retrograde paths of reentrant loop, and typical AVNRT could be induced. During effective ablation there was no junctional rhythm. In some special cases, modification of atrioventricular node should not only rely on the junctional rhythm to determine the ablation effect, but also on the time of cardiac electrophysiological examination, as such to avoid the severe complication of atrioventricular block caused by excessive ablation.

## INTRODUCTION

Many paroxysmal supraventricular tachycardia are due to atrioventricular nodal reentry, and typically, irritation conduct slowly in antegrade path and fast in retrograde path. Patients suffered from this kind of tachycardia always complain with repeatedly paroxysmal palpitation, and with sudden ending or beginning symptoms. Modification of atrioventricular node by radiofrequency catheter ablation is the most effective method to cure this disease. During this course, operators conventionally judge the ablation effect by the occurrence of junctional rhythm, and then determine the ablation time, so as to avoid injure of atrioventricular node by excessive ablation. But in some special conditions with unconventional electrophysiological phenomena, operators will confront additional difficulties and risk during ablating, such as in the case reported below.

## CASE REPORT

**Figure 1:**
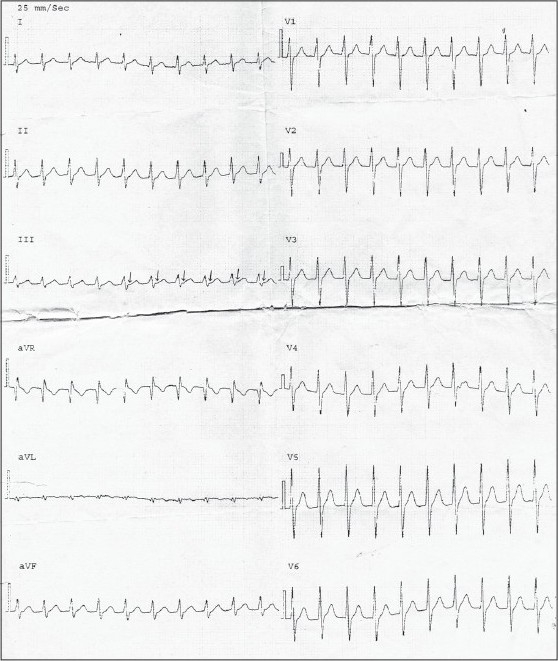
ECG during onset of paroxysmal tachycardia. Narrow QRS wave tachycardia, 167 bpm, RP = 80ms, RP < PR. Arrows direct to P wave.

A 22-year-old male patient, with paroxysmal palpitation for 10 years and aggravating shortness of breath, chest distress for 1 year, presented with tachycardia with sudden ending or beginning. There was no other disease history provided. His symptoms used to occur once a year, but more frequently in recent 1 year and were associated with chest distress and shortness of breath. He was admitted with no abnormal physical sign. Ultrasound showed normal heart structure and blood flow. Electrocardiogram (ECG) showed no abnormal appearance in sinus rhythm but displayed narrow QRS wave tachycardia when onset, 167 beats per minute (bpm) and RP = 80 ms, RP < PR [Figure 1]. These symptoms combined with the clinical history, suggested us a possible Atrioventricular nodal reentrant tachycardia.

**Figure 2:**
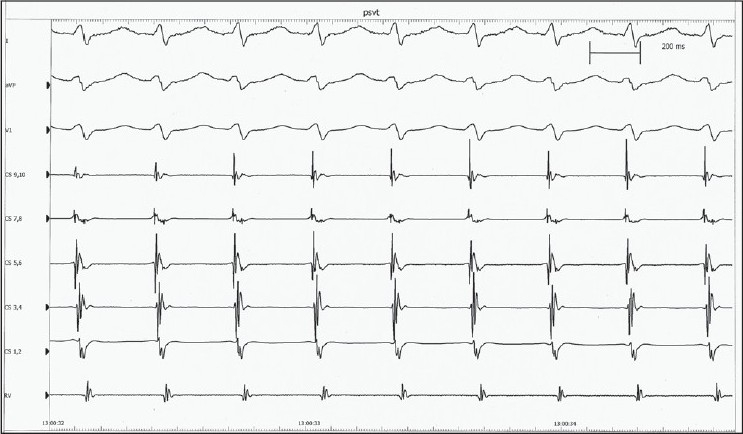
Inner ECG during tachycardia. Ventricular wave (V) and atrial wave (A) interfused at CS leads from proximal to distal. PSVT=paroxysmal supraventricular tachycardia, ECG=electrocardiogram, CS1-10=coronary sinus electrograms from distal to proximal, RV=electrogram recorded by right ventricle catheter.

**Figure 3:**
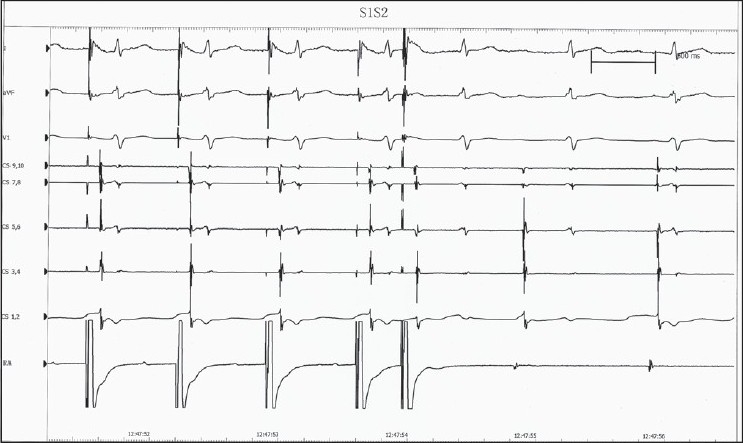
S_1_S_2_-induced atrioventricular reentrant waves, which showed the phenomenon of slow conduction in both antegrade and retrograde paths. S_1_S_2_=programmed basic stimulus coupled with cycle length decreasing extrastimulus, RA=electrogram recorded by right atrial catheter, other abbreviations as in Figure 2.

**Figure 4:**
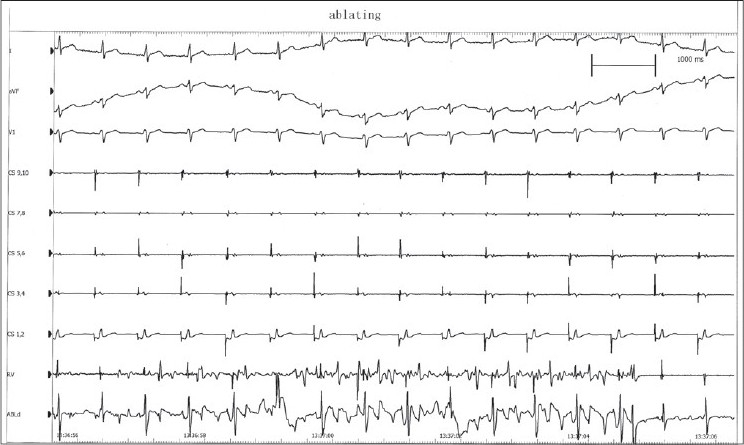
During the course of mmodification of atrioventricular node, there was no junctional rhythm. ABLd=electrogram recorded by distal leads of ablating catheter, other abbreviations as in Figure 2.

Intracardiac electrophysiological examination and ablation: mechanical irritation by catheter could induce paroxysmal supraventricular tachycardia (PSVT) during the operation, and its inner ECG shows that ventricular wave (V) and atrial wave (A) interfused at coronary sinus (CS) leads from proximal to distal [Figure 2], and it could be terminated by ventricular stimulation, showing the characteristics of atrioventricular nodal reentrant tachycardia (AVNRT). Ventricular-atrial dissociation appeared when the rate of ventricular grading frequency increment stimulation (S_1_S_1_) increased to 110 bpm; therefore, atrioventricular bypass excluded. Right atrial S_1_S_1_ did not induce tachycardia. Right atrial programmed basic stimulus coupled with cycle length decreasing extrastimulus (S_1_S_2_) induced multiple AV conduction jumps, all of their prolonged AV intervals were more than 70 ms; and induced 2-3 atrioventricular reentrant waves, which showed the phenomenon of slow conduction in both antegrade and retrograde paths [Figure 3]. Farther S_1_S_2_ scanning could induce typical AVNRT, with the characteristic of slow in antegrade and fast in retrograde conduction, and with the same rate and morphology of it in spontaneous PSVT. Examination suggested multiple reentrant paths in atrioventricular node. Subsequently, ablation of the slow conduction area of the atrioventricular node performed at several ideal target points inferior and superior to the ostium of CS, guided by inner ECG and fluoroscopic images. During this course of modification of atrioventricular node, there was no junctional rhythm [Figure 4], which always appears and been considered as the standard evaluation of the ablating effect. Normal sinus rhythm and normal atrioventricular conduction remained after modification. Repeated intracardiac electrophysiological examination: Right atrial programmed S_1_S_2_ induced no AV conduction jump phenomenon, no reentrant wave or any tachycardia; and S_1_S_1_ at right atrial and right ventricular induced no tachycardia; Wenckebach point of atrioventricular nodal conduction induced by atrial S_1_S_1_ greater than 170 bpm. Examination demonstrated the successes of the operation.

Three-month follow-up met no recurrence of tachycardia and no complication.

## DISCUSSION

PSVT due to AVNRT sometimes has unconventional manifestation.[1] Electrophysiological physicians are constantly engaged in probing its mechanism and searching for the more safe methods to perform ablation.[2–4]

From the intraoperative intracardiac electrophysiological findings and ablation results, the mechanism for the tachycardia is atrioventricular nodal reentry, slow in antegrade and fast in retrograde conduction. But the repeatable atrioventricular nodal reentries also induced by S_1_S_2_ in the examination were featured by its slow conduction in both antegrade and retrograde paths. Moreover, during effective ablating there was no junctional rhythm, which always appears and been considered as the standard evaluation of the ablating effect. This special condition displayed by these two rare electrophysiological phenomena deserves our caution.

Implications of the case: Much more judgment difficulties add to the radiofrequency ablation of AVNRT, if there is no characteristic junctional rhythm in some special complex condition which are not common, and the potential risk of severe complication also add to these cases. After a certain time of ablation by experience at chosen ideal target point guided by inner cardiac electrograms and fluoroscopic images, cardiac electrophysiological test should be performed in time to judge whether the ablation is accomplished or not, but can not only rely on the appearance of junctional rhythm to judge the effect of modification of atrioventricular node, in order to avoid the severe complication of atrioventricular block caused by excessive ablation.

In addition, if there is any correlation is unknown between the absence of junctional rhythm during ablating and the peculiarity of slow conduction in both antegrade and retrograde path of its reentrant loop before ablation.
